# Systematic review reveals sexually antagonistic knockouts in model organisms

**DOI:** 10.1002/ece3.9671

**Published:** 2022-12-28

**Authors:** Jon Alexander Harper, Edward H. Morrow

**Affiliations:** ^1^ Evolution, Behaviour and Environment Group, School of Life Sciences John Maynard Smith Building, University of Sussex Brighton UK; ^2^ Department of Environmental and Life Sciences Karlstad University Karlstad Sweden

**Keywords:** evolutionary genomics, fitness, selection‐sexual, sexual conflict

## Abstract

Sexual antagonism is thought to be an important selective force in multiple evolutionary processes, but very few examples of the genes involved are known. Such a deficit of loci could partially be explained by the lack of overlap in terminology between scientific disciplines. Following a similar review in humans, we searched systematically for studies that described genes with sexually antagonistic or sex‐opposite effects in any taxa, using terms designed to capture alternative descriptions of sexual antagonism. Despite drawing on a potentially very large pool of studies we found only eight articles, which between them described seven candidate variants, five of these were gene knockouts. In every case, the variants had net negative effects on the focal trait. One locus was independently validated between studies, but in comparison to previous data on variants in humans and the fruit‐fly, the studies generally suffered from small sample sizes, with concomitant high variance. Our review highlights the radically different effects that gene deletions can have on males and females, where the beneficial effects seen in one sex may facilitate the evolution of gene loss. We searched systematically for genetic variants with sexually antagonistic or sex‐opposite effects in any taxa. Of 2116 articles, we found seven candidate variants, five of which were gene knockouts. Our review highlights the radically different effects that gene deletions can have on males and females, where the beneficial effects seen in one sex may facilitate the evolution of gene loss.

## Graphical abstract

We searched systematically for genetic variants with sexually antagonistic or sex‐opposite effects in any taxa. Out of 2116 articles, we found 7 candidate variants, five of which were gene knockouts. Our review highlights the radically different effects that gene deletions can have on males and females, where the beneficial effects seen in one sex may facilitate the evolution of gene loss.

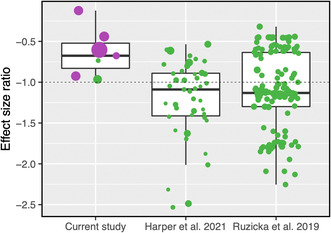

## 1 INTRODUCTION

A key step in advancing our understanding of genetic conflicts is to identify the specific loci involved (Burt & Trivers, 2008). Intralocus sexual conflict is an evolutionary conflict where alleles under positive selection in one sex experience purifying selection in the other (Parker, 1979). This sexually antagonistic (SA) selection has its origins in the evolution of separate sexes producing gametes of unequal size (Parker et al., 1972), and is thought to play an important role in the evolution of sexual dimorphism of complex traits (Lande, 1980; Rice, 1984) and gene expression (Ellegren & Parsch, 2007). It may also have broader impacts on other evolutionary processes such as speciation or extinction (Bonduriansky & Chenoweth, 2009), or contribute to sex differences in the profiles of human disease (Morrow, 2015).

While there is evidence from a number of diverse taxa that sexually antagonistic genetic variation occurs in natural and laboratory populations (Bonduriansky & Chenoweth, 2009), the identity of the specific loci involved is largely unknown (but see Barson et al., 2015; Rostant et al., 2015). In the fruit‐fly *Drosophila melanogaster*, a recent genome‐wide association study of SA variation identified a number of candidate loci that warrant independent validation (Ruzicka et al., 2019). More recently, a systematic review of the biomedical literature found around 50 specific examples of genes in humans with sex‐opposite or SA effects on traits or disease risk (Harper et al., 2021). As predicted (Morrow & Connallon, 2013), allele frequencies at these loci were higher when the net effects were positive. Crucially, none were referred to in terms of sexual conflict or sexual antagonism.

There is therefore a clear need to expand the search for SA loci to a much wider range of taxonomic groups. In this study, we use a systematic review with the aim of identifying loci with SA or sex‐opposite effects, but without directing the review to any particular taxon, though excluding humans. Given that a very broad range of traits in several laboratory and field‐based models have been investigated intensively at a genetic or genomic scale for many years, we expect to be able to find multiple examples of loci with SA or sex‐opposite effects. We also use broad search terms to help bypass the problem of discipline‐specific terminology obscuring potentially valid examples.

## 2 MATERIALS AND METHODS

We followed PRISMA guidance where possible throughout our systematic review (Moher et al., 2009). PubMed (https://pubmed.ncbi.nlm.nih.gov/) was searched for articles on August 9, 2021 with no time limit (subsequent repetitions of our search, limited to this date, yield a greater number of studies, possibly because some items are catalogued after their publication date). Studies were required to report in non‐human specific genes or genetic variants with opposite effects in the two sexes. The search terms used were designed to include papers from biomedical and ecological fields that may not have reported their findings using terms normally found within the evolutionary biology literature (Harper et al., 2021). We conducted a Boolean search, restricted to non‐human organisms, for articles that used terms in their title or abstract to describe an opposite or different effect in the two sexes or that capture this concept with alternative words for sex (“sex dependent”, “sex different”, “gender‐dependent”, “sex AND opposite”, or “gender AND opposite”, “male AND female AND opposite”), and specified a particular gene or genetic variant (AND “locus” OR “loci” OR “gene” OR “snp” OR “polymorphism” OR “variant” OR “allele”). The full search term is in the Appendix S1. The search returned 2115 papers. One additional paper from outside of the search was also considered (Schroeder et al., 2021) (Figure S1).

The abstracts of the papers from this search were then examined, and any papers that had the possibility of reporting an opposite effect of a specific genetic locus on a trait were considered for further screening. This screening produced a shortlist of 97 candidate papers (https://www.ncbi.nlm.nih.gov/sites/myncbi/1HaSgm_VzzbAzU/collections/59875782/public/). Full texts of these candidate papers were then reviewed in detail. Papers were included in the final list if they described a sex‐opposite or SA effect linked to a specific genetic locus, loci, or gene, and reported the effect to be statistically significant in both sexes (at a *p*‐value cut‐off of <.05, or with 95% confidence intervals not overlapping 1). Studies that only reported significant sex‐by‐variant effects were not automatically included unless they also satisfied the criteria above.

Reported effect sizes were used where possible. In cases where data were missing or incomplete, we extracted values from study figures using WebPlotDigitizer (version 4.5). These cases, and the figures the values were extracted from, are detailed in the Data S1. We sought information directly from the authors where these metrics were not possible to extract from the papers themselves (2 authors contacted, 1 responded, 0 responded with data). We converted all reported sex‐specific effects into a standard effect size (Cohen's *d*) quantifying the magnitude of how a given variant affects the studied trait expressed in the given sex. Specifically, Cohen's *d* was computed based on the reported descriptive statistics (*N*, mean, standard error) or by conversion from other effect sizes (Odds ratio) and test statistics (*F*‐values, *t*‐values) using formulas reported elsewhere (Borenstein, 2009; Gurevitch et al., 2013, pp. 195–206; Lajeunesse, 2013). In one case (Thomson et al., 2021), means and standard deviation were estimated from reported quartile values using the formulas described in Wan et al. (2014). We also recorded the gene name, locus, species, and the trait affected. In some cases, loci that affect multiple different traits were found. In such cases, we calculated the geometric mean of their effect sizes to account for possible pseudoreplication. All analysis was performed in R (version 4.1.3) and RStudio (build 461).

To place the fitness impacts of the SA variants identified in this study in a broader context, we began by calculating their effect size ratios (Harper et al., 2021; Morrow & Connallon, 2013), where the positive effect of a variant in one sex is divided by the negative effect in the opposite sex. An effect size ratio of −1 for a particular variant indicates that the magnitude of the positive and negative effects are perfectly balanced, net positive variants will have effect size ratios below −1, and net negative variants will have effect size ratios above −1. We then compared the effect size ratio of the variants identified in this study to those of previously identified in humans (Harper et al., 2021), and *Drosophila melanogaster* derived from sex‐specific effect sizes reported in Ruzicka et al. (2019).

Finally, we sought to find allele frequencies for the variants identified in our review to test the idea that variants with net positive effects occur at higher frequency than those with net negative effects (Harper et al., 2021; Morrow & Connallon, 2013). However, since most variants were non‐naturally occurring gene knockouts, this was not possible. We were also unable to find any naturally occurring deletions that may have been used as proxies. We were therefore unable to repeat that analysis.

## 3 RESULTS AND DISCUSSION

Our search identified eight articles that described seven variants with statistically significant sex‐opposite or SA effects on 14 traits (https://www.ncbi.nlm.nih.gov/sites/myncbi/1HaSgm_VzzbAzU/collections/61806006/public/) (Table 1). The majority (5) of variants were gene deletions (sequence removal) or knockouts (sequence inactivation or removal; in mouse, fruit‐fly, and Japanese quail), with the remaining two being naturally occurring single nucleotide polymorphisms (SNPs; in mouse and Atlantic salmon). Only one of these studies, the *VGLL* polymorphism in Atlantic salmon affecting body size and age at maturity, was previously known to us (Barson et al., 2015). This was also the only study to describe the effect in terms of sexual antagonism, reflecting the problem of discipline‐specific terminology (Harper et al., 2021). This is far fewer variants than expected given 51 variants with sex‐opposite or sexually antagonistic effects were previously found in a systematic review of humans (Harper et al., 2021). Most examples come from the mouse model, which has been intensively studied in terms of its genetics for several decades. Despite this, most loci in the mouse were experimentally induced deletions or knockouts. Although genetic associations of fitness components in other wild systems have been studied in detail, they have not apparently found examples of sexually antagonistic loci, which may reflect the numerous methodological challenges involved (Ruzicka et al., 2020).

**TABLE 1:** Genetic loci showing sexually antagonistic effects on trait expression.

Trait	Species	Gene	Locus	M effect	M effect SE	F effect	F effect SE	Reference	Figure 1B label
Distance moved in 12 h	Mouse	Mtnr1b	Deletion	−0.992	0.612	0.918	0.607	(Thomson et al., 2021)	a
Villi length	Mouse	Slfn3	KO	−4.962	1.649	1.554	0.932	(Vomhof‐DeKrey et al., 2021)	b
Muscularis externa length	Mouse	Slfn3	KO	1.971	0.995	−2.717	1.132	(Vomhof‐DeKrey et al., 2021)	b
Gastrocnemius size	Japanese Quail	MSTN	Deletion	2.500	0.504	−5.679	0.791	(Lee et al., 2020)	c
Villi length	Mouse	Slfn3	KO	−4.241	1.472	1.315	0.900	(Vomhof‐DeKrey et al., 2019)	b
Muscularis externa thickness	Mouse	Slfn3	KO	3.221	1.237	−3.188	1.230	(Vomhof‐DeKrey et al., 2019)	b
Brain injury after Hypoxy‐ischemia‐ tissue loss	Mouse	Fn14	KO	1.213	0.327	−2.214	0.375	(Kichev et al., 2018)	d
Brain injury after Hypoxy‐ischemia‐ cerebral cortex neuropathological score	Mouse	Fn14	KO	0.562	0.308	−0.586	0.309	(Kichev et al., 2018)	d
Brain injury after Hypoxy‐ischemia‐ hippocampus neuropathological score	Mouse	Fn14	KO	0.607	0.309	−1.049	0.321	(Kichev et al., 2018)	d
Brain injury after Hypoxy‐ischemia‐ thalamus neuropathological score	Mouse	Fn14	KO	0.552	0.308	−0.813	0.314	(Kichev et al., 2018)	d
Brain injury after Hypoxy‐ischemia‐ total neuropathological score	Mouse	Fn14	KO	0.608	0.309	−0.889	0.316	(Kichev et al., 2018)	d
Fertility at 29C	Drosophila	Arp53D	KO	0.313	0.663	−2.548	0.508	(Schroeder et al., 2021)	e
Maze exploration/anxiety traits	Mouse	DISC1	D453G	1.102	0.528	−1.142	0.530	(Dachtler et al., 2016)	f
Body size	Salmon	VGLL3	TOP	−2.313	0.105	1.700	0.093	(Barson et al., 2015)	g

*Note*: Effect size (Cohen's *D*). Loci are referred to as deletions or knockouts based on source terminology.

Abbreviations: F, female; KO, knockout; M, male; SE, standard error.

**FIGURE 1:**
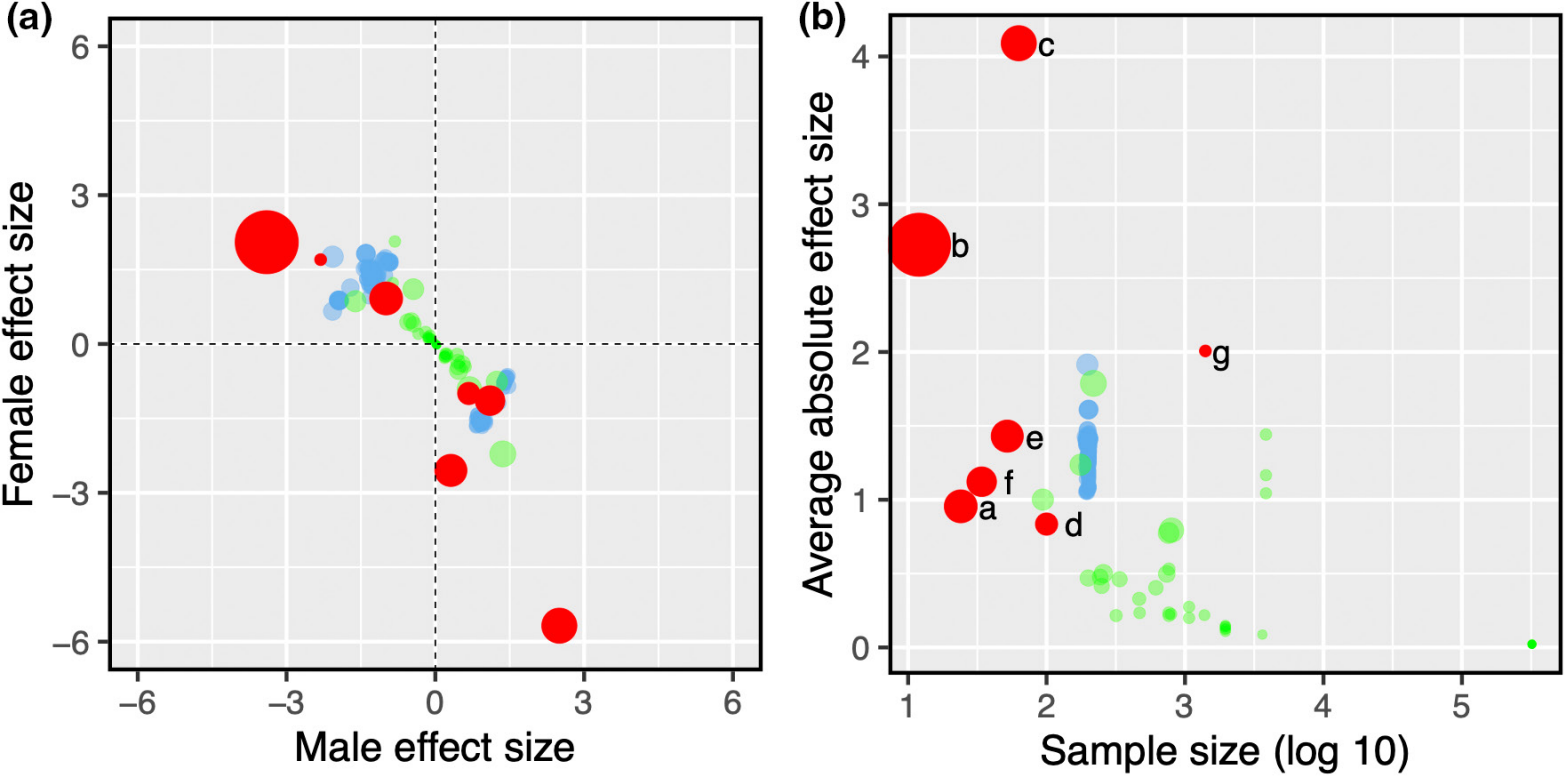
(a) Male and female effect sizes are negatively correlated in SA alleles. Effect size (Cohen's *D*) of SA alleles in males and females. Red points represent data gathered in this study. Blue points represent candidate SA *Drosophila melanogaster* alleles from Ruzicka et al. (2019). Green points represent human SA variants identified in Harper et al. (2021). Point size is scaled by variance in effect sizes. (b) Absolute effect sizes against sample size of the studies they are drawn from. Coloring of the points follows A. Individual points from the current study are also labeled: a–e synthetic variants; f and g natural variants (a: Thomson et al., 2021, Mouse; b: Vomhof‐Dekrey et al., 2021, mouse; c: Lee et al., 2020, Japanese quail; d: Kichev et al., 2018, Mouse; e: Schroeder et al., 2021, *Drosophila melanogaster*; f: Dachtler et al., 2016, Mouse; g: Barson et al., 2015, Salmon).

Overall, the effect sizes of each variant were found to be broadly similar across the two sexes (Figure 1a), though generally larger (Kruskal–Wallis, chi = 79.51, *p* = 2.2 × 10^−16^, *df* = 2) and more variable (Kruskal–Wallis, chi = 37.01, *p* = 9.19 × 10^−9^, *df* = 2) than those found in either humans (Harper et al., 2021) or the fruit‐fly (Ruzicka et al., 2019). We see two non‐mutually exclusive explanations for this pattern. First, loss‐of‐function changes like gene knockouts or deletions are likely to cause larger or more variable phenotypic effects than SNPs or smaller scale allelic variation. Alternatively, the variability and magnitude of observed in effect sizes may simply be a function of varying sample size, with smaller sample sizes being inherently noisier and therefore sometimes resulting in larger estimates. Although the variance in effect size (Kruskal‐Wallis, chi = 14.345, *p* = 1.52 × 10^−4^, *df* = 1) (Figure S2) of synthetic variants is significantly larger than naturally occurring variants this is likely confounded by differences in sample size, with average absolute effect size being larger and more variable when based on smaller samples (Figure 1b and Figure S4). Disentangling these two factors conclusively could be done with larger scale studies of the knockouts or deletions.

A clear example of a sexually antagonistic gene knockout is *Arp53D* in *D. melanogaster*, where loss of the gene significantly improved male fertility as well as reproductive success under competitive conditions (possibly mediated by faster sperm production) but was found to reduce female fertility, through a combination of maternal and zygotic effects on embryonic development (Schroeder et al., 2021). The negative effects (in females) outweighed the positive effect (in males), and so when segregating in an experimental population the knockout declines rapidly in frequency (Schroeder et al., 2021). The strength of the effect on male fertility was greater at higher rearing temperatures (25 versus 29 C) and was not significant in females at the lower temperature, though these experiments had limited sample sizes.

Our review also reveals the first validated example of a sexually antagonistic gene, the *Slfn3* (*Schlafen 3*) knockout in mice, where effects on villi length were replicated in two independent studies from the same laboratory (Vomhof‐DeKrey et al., 2019, 2021). The knockout has sex‐opposite effects on different aspects of bowel histology (villi length and muscle layer thickness; Table 1). *Schlafen 3* is thought to play a role in regulating (actually inhibiting) mucosal growth in the mouse gut during aging (Patel et al., 2009), where increased cell proliferation and decreased apoptosis in the lower digestive tract may increase the risk of cancer. It is not clear why the knockout has such different effects in the two sexes, though different genes in the *Schlafen* family can have opposing effects on gastric cancer (Al‐Marsoummi et al., 2021). The *Slfn3* knockout has been shown to increase cell proliferation and malignant melanoma cell growth in the male‐derived B16‐F1 cell line (Mavrommatis et al., 2013). An experimental next step would therefore be to look in female‐derived cell lines, or better still in vitro effects in both sexes.

**FIGURE 2:**
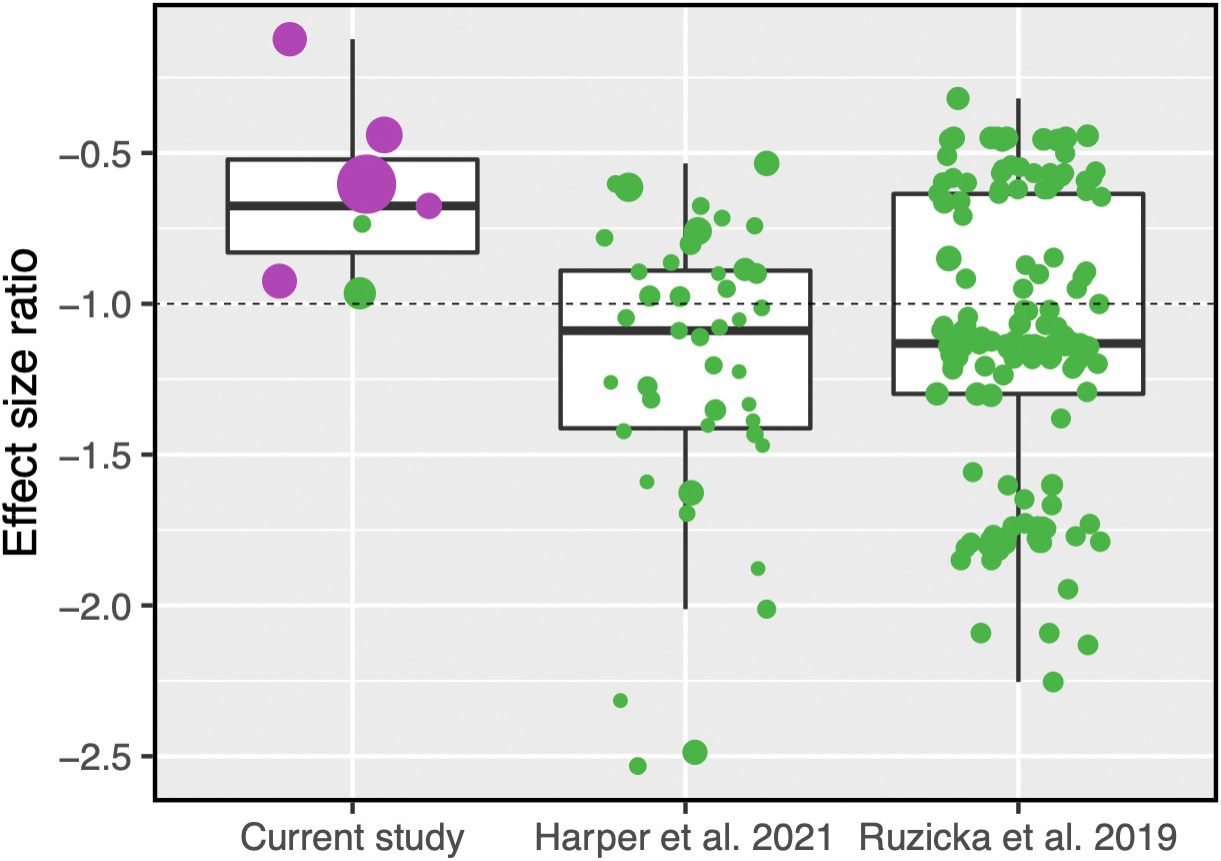
Gene knockouts have net negative effects. A comparison of effect size ratios of sexually antagonistic variants discovered in the three studies (Harper et al., 2021 human genes; Ruzicka et al., 2019 *Drosophila melanogaster*). Green points represent naturally occurring variants, purple points represent synthetic variants. The dotted line represents the threshold above which variants have net negative effects, and below net positive. Point size is scaled positively by variance in effect size.

Although we could not obtain allele frequency data, we compared the effect size ratios of the variants described here to the two other studies that describe SA candidate loci (Figure 2) (Harper et al., 2021; Ruzicka et al., 2019). No gene deletions from the current study had a net‐positive effect across the sexes (effect size ratio <−1), in stark contrast to the naturally occurring alleles which displayed both net positive and net negative effects. This is expected given that synthetic gene knockouts or deletions would more likely result in radical and deleterious effects on a phenotype compared to SNPs. It is nonetheless surprising that those kinds of changes are not universally deleterious and can apparently have positive effects in one sex. This raises the possibility that sexually antagonistic deletions could directly contribute to adaptive gene loss (Albalat & Cañestro, 2016), rather than by the much slower process of gene decay via pseudogenization that the Y‐chromosome has experienced (Bachtrog, 2013).

Our rationale for this review was that since there are numerous gonochoristic model organisms (e.g., mouse, rat, zebrafish) that have been studied in detail at a genetic level for decades, then this large literature may potentially yield numerous examples of genes with sex‐opposite or sexually antagonistic effects (as in humans, Harper et al., 2021). Clearly, this was not the case, despite using deliberately broad search terms. There are several potential explanations for this. First, sexually antagonistic genes may be rare in the species included in our review. This seems unlikely given previous evidence of sexually antagonistic genetic variation across a broad range of taxa (Bonduriansky & Chenoweth, 2009), and since there will inevitably be conflict over which alleles are favored most in males and females, even when selection does not select for divergent phenotypes between the sexes (Connallon & Clark, 2014).

Second, effects that have opposite signs in the two sexes may be discounted as false positives by investigators and go unreported, as a kind of file drawer effect. There is evidence that some investigators view those kinds of results as false positives (Wong et al., 2005). Whether this is a common problem is hard to determine. In each case, different traits are studied so it is not possible to investigate using usual meta‐analytical methods, such as funnel plots.

Model organisms may also not be studied in ways that allow SA effects to be shown. This seems quite possible, as whole gene or regional knockouts and deletions are used extensively in rodents as laboratory models of human disease, while natural standing genetic variation is rarely investigated. In wild populations of other species, the situation is different, where genomic tools have been extensively used to investigate natural variation in some systems but with the exception of Salmon this has not apparently translated into concrete examples of SA loci. There are of course significant challenges of investigating the adaptive significance of genetic variation in natural populations (Rice et al., 2005) and further difficulties in testing for SA effects (Ruzicka et al., 2020).

Finally, the studies described here generally have rather small sample sizes, which would be a significant obstacle to detecting all but the largest effects, whether SA or otherwise. In humans, genetic manipulations are not possible, therefore larger sample sizes are necessary to detect significant effects of the alleles of interest since genetic background cannot be controlled. We see small sample size regularly used in studies of non‐human models as the most likely major limiting factor for identifying genes with SA effects.

## AUTHOR CONTRIBUTIONS

**Jon Alexander Harper**: Conceptualization (equal); data curation (lead); formal analysis (lead); investigation (equal); methodology (equal); visualization (lead); writing – original draft (lead), writing – review and editing (equal). **Edward Morrow:** Conceptualization (equal); data curation (supporting); formal analysis (equal); funding acquisition (lead); investigation (supporting); methodology (supporting); supervision (lead); visualization (supporting); writing – original draft (supporting); writing – review and editing (equal).

## CONFLICT OF INTEREST

The authors declare no conflict of interest.

## Supporting information


Appendix S1.

Data S1.

## Data Availability

Shortlist can be found at: https://www.ncbi.nlm.nih.gov/sites/myncbi/1HaSgm_VzzbAzU/collections/59875782/public/. Selected papers can be found at: https://pubmed.ncbi.nlm.nih.gov/collections/61806006/?sort=pubdate. Data files and code can be found at: https://doi.org/10.5281/zenodo.6782329.
