# Supplementary material for: Systematic review reveals sexually antagonistic knockouts in model organisms
Source: Ecol Evol. 2022 Dec 28;12(12):e9671. doi: 10.1002/ece3.9671 (PMC9798040; doi:10.1002/ece3.9671)
Supplement: Supplementary file 1 — Appendix S1. [file ECE3-12-e9671-s001.docx]

# Supplementary material

Search term:

((((((((((((((((((((((((((((((((((((((((gender[Title/Abstract] AND opposite[Title/Abstract] AND locus[Title/Abstract]) OR (gender[Title/Abstract] AND opposite[Title/Abstract] AND loci[Title/Abstract])) OR (gender[Title/Abstract] AND opposite[Title/Abstract] AND gene[Title/Abstract])) OR (gender[Title/Abstract] AND opposite[Title/Abstract] AND snp[Title/Abstract])) OR (gender[Title/Abstract] AND opposite[Title/Abstract] AND polymorphism[Title/Abstract])) OR (gender[Title/Abstract] AND opposite[Title/Abstract] AND variant[Title/Abstract])) OR (gender[Title/Abstract] AND opposite[Title/Abstract] AND allele[Title/Abstract])) OR (sex[Title/Abstract] AND opposite[Title/Abstract] AND locus[Title/Abstract])) OR (sex[Title/Abstract] AND opposite[Title/Abstract] AND loci[Title/Abstract])) OR (sex[Title/Abstract] AND opposite[Title/Abstract] AND gene[Title/Abstract])) OR (sex[Title/Abstract] AND opposite[Title/Abstract] AND snp[Title/Abstract])) OR (sex[Title/Abstract] AND opposite[Title/Abstract] AND polymorphism[Title/Abstract])) OR (sex[Title/Abstract] AND opposite[Title/Abstract] AND variant[Title/Abstract])) OR (sex[Title/Abstract] AND opposite[Title/Abstract] AND allele[Title/Abstract])) OR (sex dependent[Title/Abstract] AND locus[Title/Abstract])) OR (sex dependent[Title/Abstract] AND loci[Title/Abstract])) OR (sex dependent[Title/Abstract] AND gene[Title/Abstract])) OR (sex dependent[Title/Abstract] AND snp[Title/Abstract])) OR (sex dependent[Title/Abstract] AND polymorphism[Title/Abstract])) OR (sex dependent[Title/Abstract] AND variant[Title/Abstract])) OR (sex dependent[Title/Abstract] AND allele[Title/Abstract])) OR (sex different[Title/Abstract] AND locus[Title/Abstract])) OR (sex different[Title/Abstract] AND loci[Title/Abstract])) OR (sex different[Title/Abstract] AND gene[Title/Abstract])) OR (sex different[Title/Abstract] AND snp[Title/Abstract])) OR (sex different[Title/Abstract] AND polymorphism[Title/Abstract])) OR (sex different[Title/Abstract] AND variant[Title/Abstract])) OR (sex different[Title/Abstract] AND allele[Title/Abstract])) OR (gender-dependent[Title/Abstract] AND locus[Title/Abstract])) OR (gender-dependent[Title/Abstract] AND loci[Title/Abstract])) OR (gender-dependent[Title/Abstract] AND gene[Title/Abstract])) OR (gender-dependent[Title/Abstract] AND snp[Title/Abstract])) OR (gender-dependent[Title/Abstract] AND polymorphism[Title/Abstract])) OR (gender-dependent[Title/Abstract] AND variant[Title/Abstract])) OR (gender-dependent[Title/Abstract] AND allele[Title/Abstract])) OR (male[Title/Abstract] AND female[Title/Abstract] AND opposite[Title/Abstract] AND locus[Title/Abstract])) OR (male[Title/Abstract] AND female[Title/Abstract] AND opposite[Title/Abstract] AND loci[Title/Abstract])) OR (male[Title/Abstract] AND female[Title/Abstract] AND opposite[Title/Abstract] AND gene[Title/Abstract])) OR (male[Title/Abstract] AND female[Title/Abstract] AND opposite[Title/Abstract] AND snp[Title/Abstract])) OR (male[Title/Abstract] AND female[Title/Abstract] AND opposite[Title/Abstract] AND polymorphism[Title/Abstract])) OR (male[Title/Abstract] AND female[Title/Abstract] AND opposite[Title/Abstract] AND variant[Title/Abstract])

**Figure S1. PRISMA flow diagram for systematic review of non-human sexually antagonistic variants**

**Figure S2. Synthetic variants have greater variance in effect size than natural variants.** Comparison of variance of synthetic and natural SA variants. All synthetic variants are from the current study. Natural variants include data from this study, Harper et al., (2021) and Ruzicka et al. (2019).

**Figure S3. Natural and synthetic variant effect sizes.** Average absolute effect sizes between males and females of SA variants. All synthetic variants are from the current study. Natural variants include data from this study, Harper et al., (2021) and Ruzicka et al. (2019).


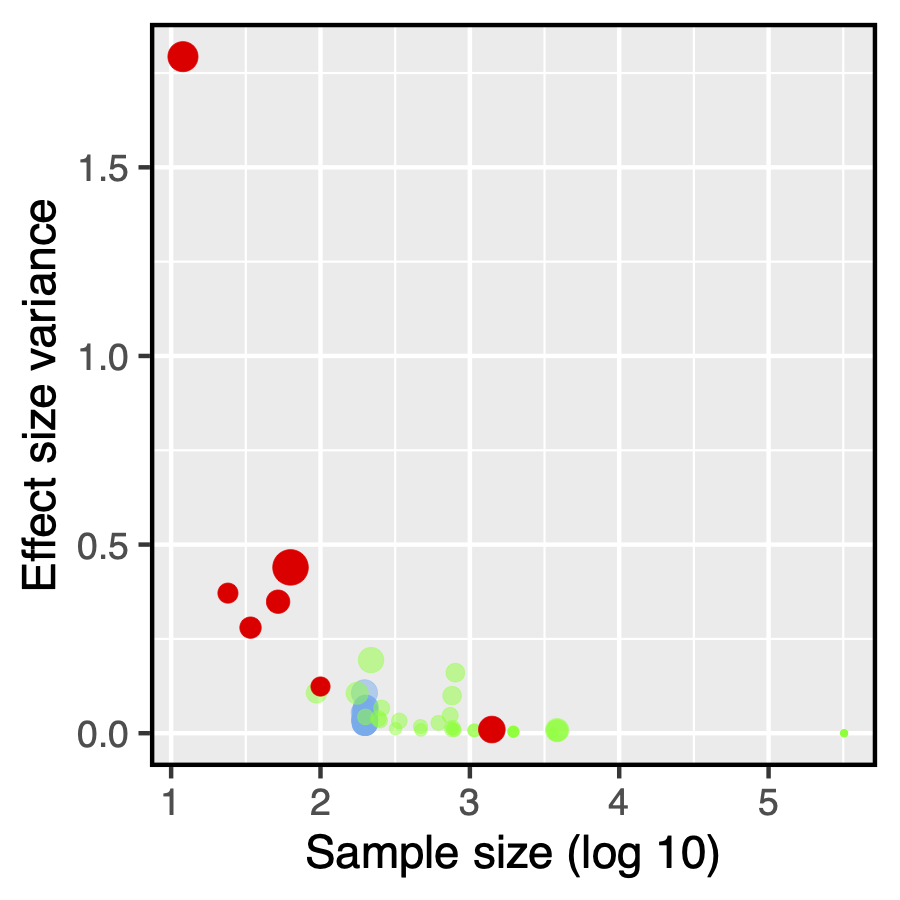


**Figure S4. Studies with larger sample sizes have lower variance in effect size.** Variance of effect sizes against the sample size of the study. Points are scaled with their effect size; larger points have higher effect sizes. Green points represent data from Harper et al., (2021) and blue points represent data from Ruzicka et al. (2019).
